# Development and validation of a novel necroptosis-related score to improve the outcomes of clear cell renal cell carcinoma

**DOI:** 10.3389/fgene.2022.967613

**Published:** 2022-09-12

**Authors:** Ji Chen, Qiqi Tao, Zhichao Lang, Yan Jin, Guanqi Chen, Xinling Li, Zhixian Yu, Yeping Li

**Affiliations:** ^1^ Key Laboratory of Diagnosis and Treatment of Severe Hepato-Pancreatic Diseases of Zhejiang Province, The First Affiliated Hospital of Wenzhou Medical University, Wenzhou, China; ^2^ Department of Urology, The First Affiliated Hospital of Wenzhou Medical University, Wenzhou, China

**Keywords:** necroptosis, clear cell renal cell carcinoma, prognosis, subtypes, survival

## Abstract

Necroptosis has been indicated as a key regulator of tumor progression. However, the prognostic regulatory role of necroptosis in clear cell renal cell carcinoma (ccRCC) needs to be further investigated. In this study, necroptosis-related subtypes were identified by mining the public cohort (*n* = 530) obtained from The Cancer Genome Atlas. By applying Principal Component Analysis (PCA), the necroptosis-related scores (N-Score) were developed to assess the prognosis procession of ccRCC. The results were further validated by an external clinical cohort (*n* = 116) obtained from the First Affiliated Hospital of Wenzhou Medical University. It has been found that N-Score could precisely distinguish the prognostic outcomes of patients as an independent risk factor (Hazard ratio = 4.990, 95% confidence interval (CI) = 2.007–12.403, *p* < 0.001). In addition, changes in N-Score were associated with differences in tumor mutational burden as well as immune infiltration characterization. Moreover, higher N-Scores were also correlated significantly molecular drug sensitivity and stronger immune checkpoint activity. Notably, the prognosis of ccRCC could be effectively guided by combining the N-Scores and external clinical indicators. In conclusion, N-Scores could be served as a robust and effective biomarker to improve the prognosis outcomes and targeted therapy of ccRCC.

## Introduction

Renal cell carcinoma (RCC) is one of the most common urological cancers worldwide, among which 75% of RCC death cases are clear cell renal cell carcinoma (ccRCC) (Hsieh et al., 2017; Jonasch et al., 2021). Features of ccRCC with high prevalence and poor advanced prognosis could be largely attributed to tumor progression and undertreatment (Jonasch et al., 2021; Wei et al., 2021). ccRCC preferentially invades perinephric adipose tissue (PAT) at an early stage, which is associated with its poor advanced prognosis (Bedke et al., 2009). The American Joint Commission on cancer (AJCC) classification is a routine tool for assessing the staging of ccRCC. However, considering the special molecular biology of ccRCC, the optimal clinical care is decided mainly with the aid of clinicopathological staging. Hence, imperfections in staging and tumor progression assessment may lead to potential overtreatment or undertreatment. Actually, the overall prognosis of ccRCC is still not promising, and new indicators are urgently needed to precisely assess the tumor procession and predict the prognosis. Currently, surgical resection still remains the mainstay of treatment for localized tumors of ccRCC (Sahni et al., 2016). Accumulating studies have revealed that emerging targeted agents contribute to improving therapies for ccRCC (Wettersten et al., 2017; Choueiri and Kaelin, 2020). However, due to the complex intra- and inter-tumoral disease heterogeneity of ccRCC, conventional surgical treatments and medical therapies have shown little efficacy. The emerging evidence has also reported that immunotherapy using immune checkpoint inhibitors (ICIs) holds promise as a new opportunity for targeted therapy in ccRCC (Srivastava et al., 2022). Moreover, PD-L1 and CD47 have been found to mediate ICI immunotherapy to improve the outcome of ccRCC (Kong et al., 2021; Jiang et al., 2022). But unfortunately, the improvement of RCC prognosis by conventional immunotherapy is not satisfactory, either (Sobottka et al., 2022).

Necroptosis, as a form of programmed cell death, is characterized by loss of membrane integrity and release of intracellular contents (Fritsch et al., 2019; Zhou et al., 2020). Through critical molecular switches such as caspase-8, necroptosis plays a tremendous role in multiple tissue structural injuries and disease processes (Yuan et al., 2019; Zhang et al., 2020). Increasing studies have demonstrated that the biological and therapeutic responses of multiple cancers are further influenced by necroptosis (Cao and Tait, 2019; Chen et al., 2021). Induced by local or systemic treatments, necroptosis also effectively inhibits tumor proliferation, invasion, and metastasis (Koren and Fuchs, 2021). Recently, it has been reported that necroptosis can remarkably increase reactive oxygen species (ROS) levels in RCC, thereby inhibiting tumor growth processes (Wang et al., 2021). Meanwhile, AI-Lamki et al. found that the cell cycle of ccRCC is dramatically altered by increasing the level of necroptosis and receiving TNF receptor transduction (Al-Lamki et al., 2016). Unfortunately, these studies of necroptosis in ccRCC are still limited in the molecular level. It is urgently to address the role of necroptosis in ccRCC in clinical and prognostic terms.

As a highly heterogeneous cancer, developing highly effective biomarkers for ccRCC is extremely difficult (Koh et al., 2021). Actually, an ideal biomarker needs to be developed with the aid of vast gene expression profiling datasets and rigorous clinical cohorts. Furthermore, the biomarker should effectively assess the biological processes and prognosis of ccRCC. Meanwhile, the ideal biomarker should also robustly cross all patients. At present, it has been found that apoptosis-related biomarkers may effectively improve the outcomes of ccRCC (Bai et al., 2022; Wu et al., 2022). However, there are still no similar studies exploring necroptosis-related biomarkers in ccRCC.

In the present study, we attempted to apply necroptosis-related genes (NRGs) to develop a novel necroptosis-related scores (N-Score) to improve the prognostic outcomes of ccRCC. The reliability and deep clinical value of N-Scores were further validated in an external clinical cohort. Meanwhile, we aimed to explore the novel molecular agents and ICI treatments to improve the targeted therapy for ccRCC. In conclusion, this work contributed to optimizing precise treatment and prognosis prediction for ccRCC.

## Materials and methods

### Data preparation

In total, 530 ccRCC patients, containing their entire RNA-seq profiles and clinical characteristics [overall survival (OS) time, OS status, age, tumor grade, T stage, N stage, and M stage] were obtained from The Cancer Genome Atlas (TCGA) (https://portal.gdc.cancer.gov/). The RNA-seq raw read count was converted to transcripts per kilobase million (TPM) and further log-2 transformed. They were enrolled as the TCGA cohort to identify necroptosis-related clusters (N-Clusters) and develop the necroptosis-related scores (N-Scores). For further validating the results and exploring the clinical implications of N-Scores, a total of 116 patients were collected from the First Affiliated Hospital of Wenzhou Medical University (FAHWMU) as the external clinical cohort. The collection of this cohort was reviewed and approved by the human research ethics committee of the First Affiliated Hospital of Wenzhou Medical University. All patients/participants provided their written informed consent to participate in this study.

Approved by the ethics committee of the First Affiliated Hospital of Wenzhou Medical University, this study retrospectively collected the clinical data of patients who underwent renal surgery in the Department of Urology of the First Affiliated Hospital of Wenzhou Medical University. Among the clinical characteristics in the FAHWMU cohort, the histopathological variables (including tumor size, Platelet, Calcification, Hemoglobin, Neutrophils, KPS score, and WHO/ISUP grade) were determined by consensus between two professional pathologists. Based on the 8th edition of the AJCC Staging Manual, the TNM stage for each ccRCC patient was assessed. In addition, age and gender were included as the demographic characteristics. The specific clinical characteristics in the FAHWMU cohort were listed in Table. 1.

**TABLE 1:** Clinical characteristics for ccRCC patients in the FAHWMU cohort.

	FAHWMU cohort (*n* = 116)
Age, years	50.89 ± 10.47
Gender	
Male	72 (62.1%)
Female	44 (37.9%)
TNM stage	
I	63 (54.3%)
II	23 (19.8%)
III	25 (21.6%)
IV	5 (4.3%)
WHO/ISUP grade	
I	40 (34.5%)
II	46 (37.1%)
III	23 (19.8%)
IV	7 (6.0%)
Tumor size, cm	3.76 ± 0.94
KPS score	
≥80%	105 (90.5%)
<80%	11 (9.5%)
Serum calcium	
≤10.2 mg/dl	84 (72.4%)
>10.2 mg/dl	32 (27.6%)
Platelet	
≤normal level	97 (83.6%)
>normal level	19 (16.4%)
Calcification	
−	95 (81.9%)
+	21 (18.1%)
Hemoglobin	
≥120 g/L	71 (61.2%)
<120 g/L	45 (38.8%)
Neutrophils	
≤7 × 10^9^/L	94 (81.0%)
>7 × 10^9^/L	22 (19.0%)

The eight necroptosis-related genes (NRGs) (FADD, FAS, FASLG, MLKL, RIPK1, RIPK3, TLR3, and TNF) used in this study were obtained from MSigDB database (http://www.gsea-msigdb.org/gsea/msigdb/). RIPK3, RIPK1, and FADD are key regulatory medicators that drive the assembly of inflammatory body and mediate necroptosis (Lee et al., 2021a). FAS, FASLG, and MLKL can lead to lysosomal membrane permeabilization and cathepsin release to the cytoplasmic matrix, which induces necroptosis formation (Sharapova et al., 2018). TLR3 and TNF play a key role in the necroptosis mediated by the receptor-interacting protein (Taft et al., 2021).

Raw data of cancer mutations (including mutated genes, mutation frequency, mutation types, etc.), were enrolled from TCGA database (https://portal.gdc.cancer.gov/). Somatic mutation analysis implemented in R package “matfool” was employed to obtain the tumor mutation burden (TME) score for individual ccRCC patient (Krøigård et al., 2016; Addeo et al., 2019). The imvigor210 cohort was downloaded online with the R package IMvigor210CoreBiologies.

### Quantitative real-time PCR

The total RNA from the renal tissues of the FAHWMU cohort was extracted using TRIzol reagent. The mRNA was then reverse transcribed into cDNA using ribo SCRIPTTM reverse transcription kit. The expression levels of mRNA were calibrated with glyceraldehyde-3-phosphate dehydrogenase (GAPDH). SYBR Green master mix was added, and real-time PCR was carried out using a 7500 rapid quantitative PCR system (Applied Biosystems, United States). The CT value of each well was recorded, and the relative quantification of the amplified products was performed using the 2^−ΔCt^ method.

### Enrichment analysis

Based on the R package GSVA, Single-sample gene set enrichment analysis (ssGSEA) was performed to calculated the relative infiltration characteristics of 16 immune cells and 13 immune related functions in the TCGA cohort. The relative content of 22 immune cells were quantified according to the CIBERSOFT algorithm. Gene Ontology (GO) enrichment analysis was employed by the R package “GOplot”. Kyoto Encyclopedia of Genes and Genomes (KEGG) enrichment analysis was performed through DAVID database [DAVID Functional Annotation Bioinformatics Microarray Analysis (ncifcrf.gov)]. According to the Genomics of Drug Sensitivity in Cancer (GDSC; https://www.cancerrxgene.org) database, the response of patients to possible molecular agents was predicted (Geeleher et al., 2014).

### Principal components analysis

According to the expression profiles of NRGs, we defined genes with similar expression types as individual modules using linear transformation. Then, PCA network was generated by transforming gene expression modules into a multi-dimensional network. The dimensions of each principal component were compressed based on the input of expression modulates and output across prognosis. Subsequently, the continuous data dimension reduction was performed until the expression module within the two-dimensional space could achieve the best prediction of prognosis. Thus, the N-score was obtained. For distinguishing the high N-Score group and the low N-Score group, we used the surv-cutpoint function in the K-M survival analysis to select the optimal cutoff values. The surv-cutpoint function was mainly based on the R package “survminer”. According to the optimal cutoff value, patients were assigned into the high N-Score group and the low N-Score group.

### Consensus clustering

Consensus clustering was performed based on the R package “ConsensusClusterPlus” to select optimal N-clusters (Kiselev et al., 2017). To determine the optimal k value, the resampling method was used to sample a certain sample data set. It specified the number of clusters k, and calculated the plausibility under different cluster numbers. With the consensus index value from 0.1 to 0.9, the k value with the smallest cumulative distribution function (CDF) slope was considered the optimal value to separate the clusters.

### Statistical analysis

All statistical analyses were performed in the R software [R: The R Project for Statistical Computing ( r-project.org, version 4.1.3)]. For all clinical cohorts, differential analysis was applied by Wilcoxon test to ensure that it did not depend on the probability distribution belonging to any specific parameter. The receiver operating characteristic (ROC) curve implemented *via* R package “timeROC”. The Kaplan-Meier survival curve, nomogram, calibrate curves, and decision curve analysis (DCA) were constructed using R package “rms”. Statistical *p*-values were subjected to two tailed tests, and *p* < 0.05 was considered as significance.

## Results

### Development of necroptosis-related subtypes and relevant necroptosis scores

**FIGURE 1:**
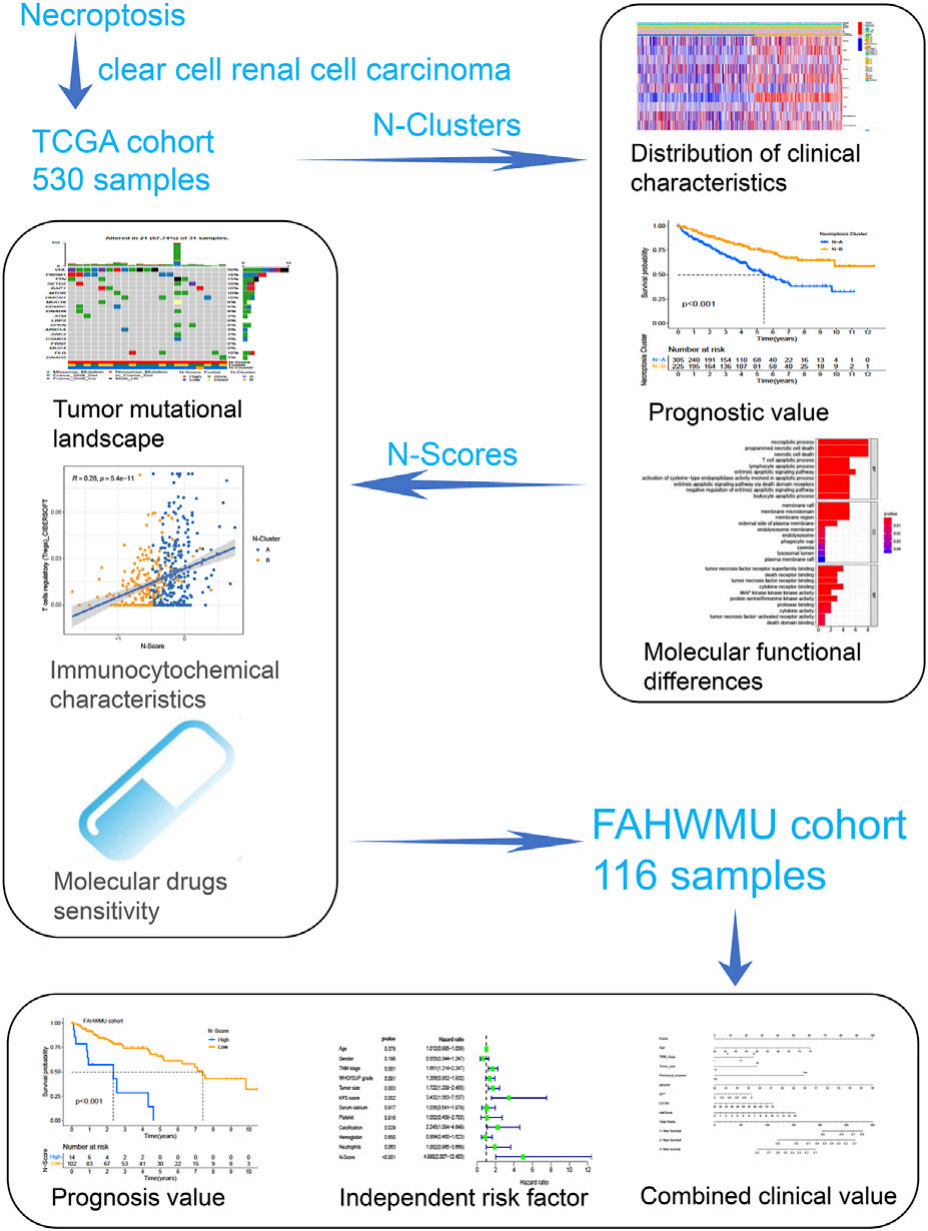
The overall design of this study.

**FIGURE 2:**
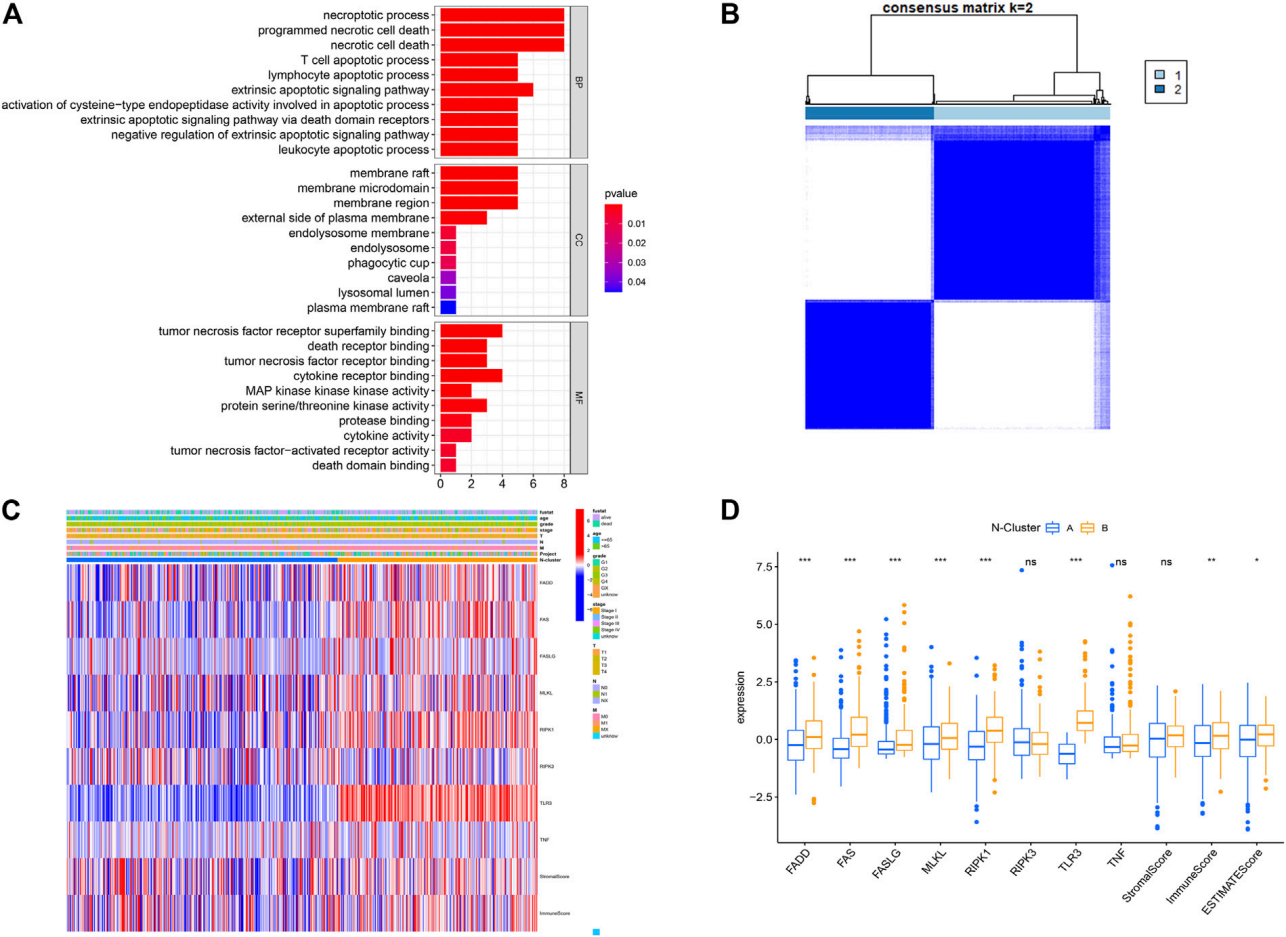
Construction of Necroptosis related cluster (N-Cluster). **(A)** Gene Ontology (GO) analysis for eight Necroptosis-related genes (NRGs) (*p* < 0.05, *q* < 0.05). **(B)** Consensus clustering analysis based on the expression levels of eight NRGs. **(C)** Complex heat map representing the difference in NRGs expression, clinical characteristics, Immune Score, Stromal Score between N-Clusters. **(D)** Boxplot displaying the differences in NRG expression between N-Clusters (*: *p* < 0.05, **: *p* < 0.01, and ***: *p* < 0.001).

**FIGURE 3:**
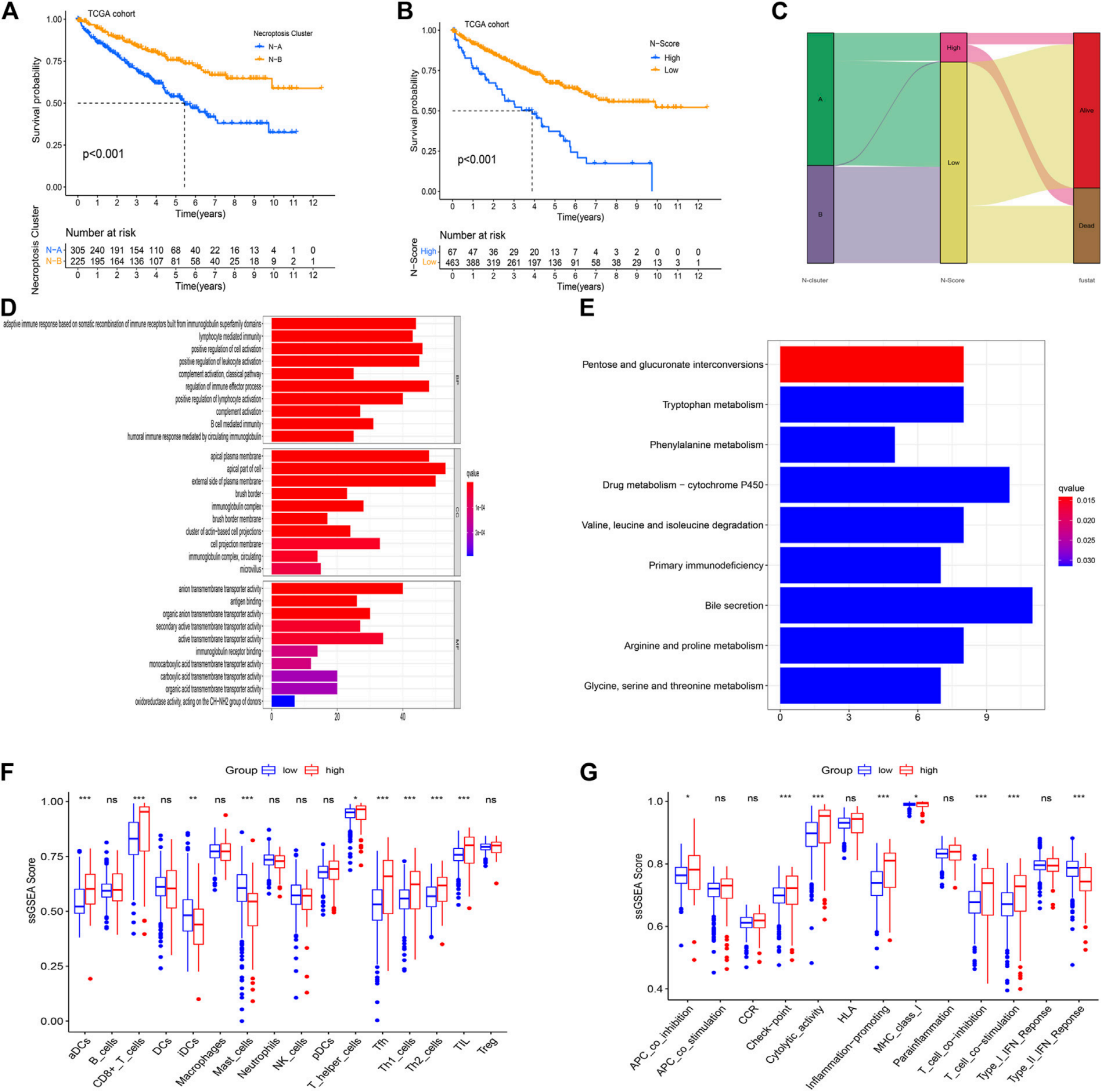
Enrichment analysis for the necroptosis-related score (N-Score). **(A)** Kaplan-Meier (K-M) survival curve shows that the effect of N-Clusters on overall survival (OS) in the TCGA cohort (*p* < 0.001). **(B)** Kaplan-Meier (K-M) survival curve shows that the effect of N-Scores on overall survival (OS) in the TCGA cohort (*p* < 0.001). **(C)** Sankey diagram shows the correlation between N-Cluster, N-Score and OS status. **(D)** GO analysis for differential expressed genes (DEGs) between low N-Score group and high N-Score group. **(E)** Kyoto Encyclopedia of Genes and Genomes (KEGG) analysis for DEGs between N-Score groups. **(F,G)** The difference for ssGSEA scores of 16 immune cells **(F)** and 13 immune related functions **(G)** between N-Score groups (*: *p* < 0.05, **: *p* < 0.01, and ***: *p* < 0.001).

As shown in Figure 1, this work could be summarized from the following aspects: 1) Identification of N-Clusters; 2) Development of N-Scores; 3) N-Scores can be served as an independent clinical factor; 4) N-Scores corresponded with TMB and immune infiltration; 5) N-Scores contributed to molecular drugs therapy and ICI treatment for ccRCC; 6) N-Scores showed the robust clinical value in the external FAHWMU cohort. Figure 2A displayed the enrichment of molecular biological functions for eight NRGs. It could be found that expect for necroptosis-related process, NRGs were mainly enriched in the immune pathway mediated programmed apoptosis (e.g., T cell apoptotic process, lymphocyte apoptotic process, and extrinsic apoptotic signaling pathway, etc.). According to the gene expression profiles of NRGs, consensus clustering was performed in all ccRCC samples to select the optimal k value from 2 to 9 (Supplementary Figure S1A). The cumulative distribution function (CDF) curves of the consensus clustering showed that the optimal subtypes were obtained when k value = 2 (Supplementary Figure S1B). Thus, the N-Clusters were determined (Figure 2B). The distribution of clinical characteristics, ESTIMATE scores, and NRGs expression levels was shown in Figure 2C. The ESTIMATE scores (Stromal score, Immune score, and ESTIMATE score) were inferred by ESTIMATE algorithm as indicators to predict the level of infiltrating stromal and immune cells. As indicated in Figure 2D, the Immune score and ESTIMATE score for patients in N-Cluster B were significantly higher compared to patients in N-Cluster A (*p* < 0.05). Our results demonstrated that N-Cluster B had higher levels of tumor purity and immune infiltration, which was closely associated with the increase of immune responses induced by necroptosis. Figure 3A displayed that the patients in N-Cluster B had better OS compared to patients in N-Cluster A (*p* < 0.001).

Using PCA analysis, the difference in the dimensionality of NRGs between N-Clusters was analyzed. Thus, we performed the dimension reduction analysis to determine a novel N-Scores. K-M survival curve revealed that N-Scores can positively predict the prognosis for ccRCC patients (Figure 3B, *p* < 0.001). Figure 3C revealed the relationships among N-Clusters, N-Scores and OS status. It was found that N-Scores were remarkably correlated with better OS status. Based on the comparison of cellular biological functions and cellular pathways between different N-Scores groups, the GO and KEGG enrichment analyses were performed. It was found that high N-Scores group was significantly enriched in a large number of immune response functions and membrane disrupting functions (e.g., adaptive immune response based on somatic recombination of immune receptors built from immunoglobulin superfamily domains, regulation of immune effector process, apical plasma membrane and external side of plasma membrane, etc.) (Figure 3D). Meanwhile, similar cellular pathways (e.g., Primary immunodeficiency, Pentose and glucuronate interconversions and Drug metabolism-cytochrome P450, etc.) were also enriched according to the KEGG enrichment analysis (Figure 3E). The change of cellular biological functions and pathways was associated with the basic bioprocess changes in necroptosis, indicating the clinical typing value of the N-Scores from the side. As indicated in Figure 3F, the relative content of several immune cells (aDCs, CD8^+^ T cells, iDCs, Mast cells, T helper cells, Tfh, Th1 cells, Th2 cells, and TIL) for patients with high N-Scores was significantly higher. In addition, the proportion of certain immune-related pathways (APC co-inhibition, Check point, Cytolytic activity, Inflammation promoting, MHC class I, T cell co-inhibition, T cell co-stimulation, Type II IFN Response) showed the similar results (Figure 3G).

### N-scores could be served as a robust risk factor

**FIGURE 4:**
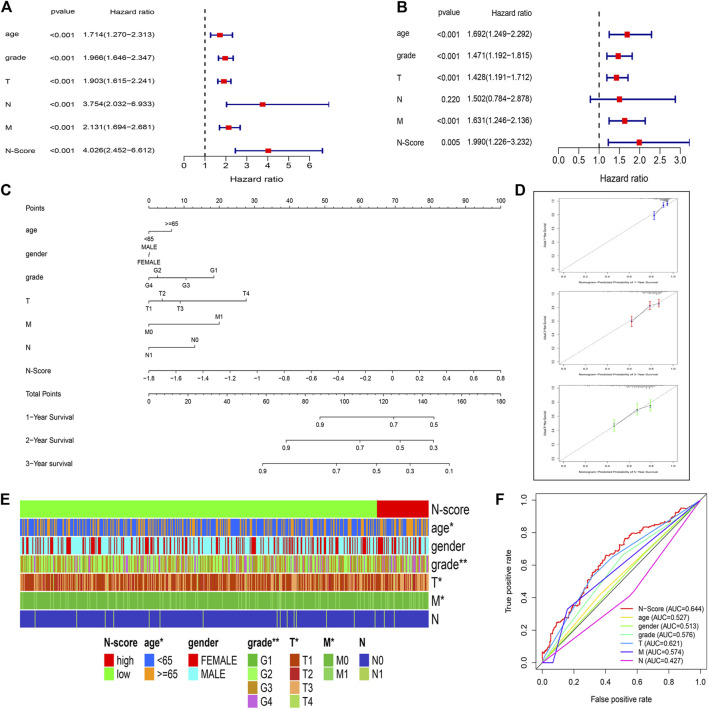
N-Score could be served as an independent clinical factor. **(A,B)** Forest plots display the Univariate Cox and multivariate Cox analysis for clinical characteristics (age, gender, T stage, N stage, and M stage) and N-Score. **(C)** Nomogram combining the N-Score and clinical characteristics (age, gender, T stage, N stage, and M stage). **(D)** Calibrate curves of the nomogram in the 1st, 3rd, and 5th years. **(E)** Heat map shows the increase of N-Score is correlated with the increase of clinical characteristics (age, gender, T stage, N stage, and M stage). **(F)** Time independent receiver operating characteristic (ROC) curve for N-Score and clinical characteristics in the 3rd years.

To determine whether the N-Scores could be served as an independent risk factor, the univariate Cox analysis and multi-variate Cox analysis were co-performed. It was found that age, tumor grade, T stage, N stage, M stage, and N-Score can independently predict the OS as the risk factors (Figure 4A). In addition, the combined value of these factors was also excellent in the multi-variate Cox analysis (Figure 4B). To better combine these indicators to precisely predict prognosis for ccRCC, a novel clinical nomogram was constructed (Figure 4C). The N-Score showed higher sensitivity and prediction efficiency than other clinical characteristics. Validated by the calibrate curves in the 1st, 2nd, and 3rd years, the nomogram can be a robust tool in the prognosis prediction of ccRCC (Figure 4D). The DCA analysis further demonstrated that the role of N scores in clinical decision-making is efficient in the 1st, 2nd, and 3rd years (Supplementary Figures S2A–C). The distribution of clinical characteristics with the increase of N-Score was shown in Figure 4E. It could be found that the change of N-Score could be significantly correlated with age, tumor grade, T stage, and M stage. In addition, the 1-year ROC curve displayed that the clinical diagnostic value of N-Score was also better than other clinical characteristics (Figure 4F).

### Implications of N-scores to tumor mutation burden

**FIGURE 5:**
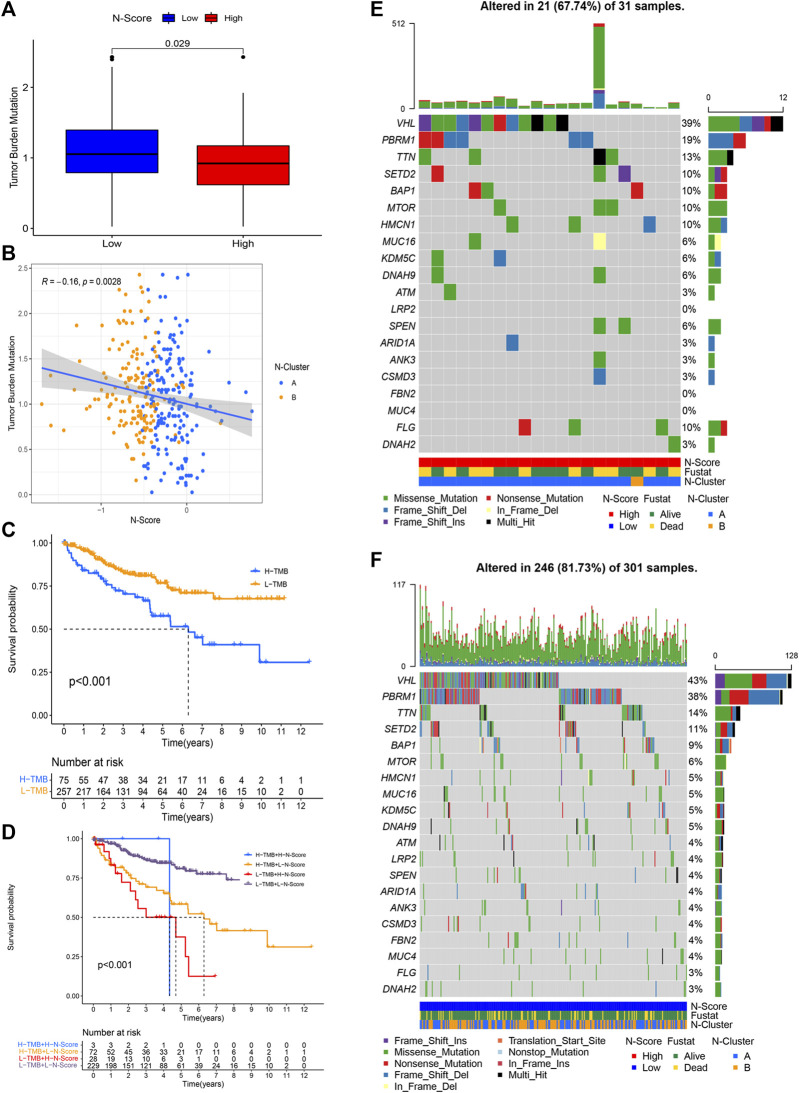
Correlation between tumor mutation burden (TMB) and N-Score. **(A)** Boxplot shows patients in the low N-Score group have higher TMB value. **(B)** Correlation between N-Score, N-Cluster, and TMB values. **(C)** K-M curve displays the OS is worse in the high TMB value groups. **(D)** K-M curve reveals the individual effect of TMB values and N-Scores on prognosis. **(E,F)** Oncoplots displays the correlation of mutant genes, N-Clusters and OS status in the high N-Score group **(E)** and low N-Score group **(F)**.

Based on the R package matfools and methods described above, TMB score for individual ccRCC patient was calculated. Correlation plots of TMB and N-Scores showed that samples with lower TMB had paradoxically higher N-Scores (Figures 5A,B, *p* = 0.0028, *R* = −0.16). Figure 5C showed that the OS rate of patients with high TMB was obviously worse than that of patients with low TMB (*p* < 0.001). K-M survival curve showed that the OS rate of patients with low TMB and low N-Scores was significantly better than that of other patients (Figure 5D, *p* < 0.001). In Figure 5D, the statistical significance of High TMB and High N-score group was lost due to the scarce number (*n* = 3). Additionally, the oncoplots demonstrated the differences of mutated genes, mutation frequency, mutation types and OS status between N-Scores groups (Figures 5E,F). It could be indicated that low N-Scores was related with high mutation frequency and mutation strength. Moreover, most of the mutated genes were tumor suppressors, which probably contributed to the lower TMB. These results were consistent with the previous correlation of N-Scores and TMB.

**FIGURE 6:**
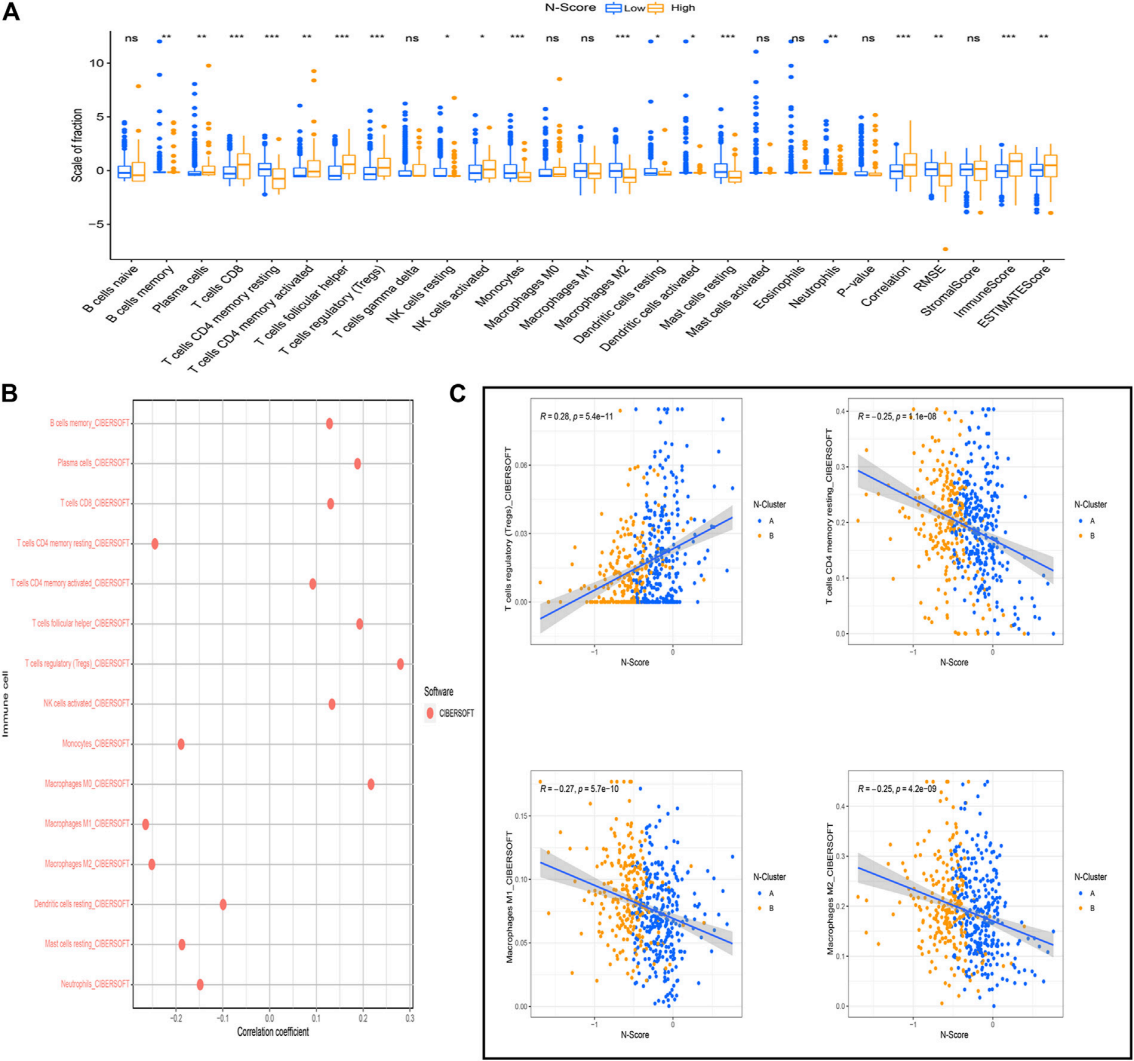
Association between N-Score and immune cells. **(A)** The difference of the scale of immune cells calculated by the CIBERSOFT algorithm between N-Score groups (*: *p* < 0.05, **: *p* < 0.01, and ***: *p* < 0.001). **(B)** Correlation between N-score and the scale of immune cells calculated by the CIBERSOFT algorithm. **(C)** Scatter diagrams reveals the correlations between N-Scores and the significant immune cells with highest coefficient (T cells regulatory (Tregs), T cells CD4 memory resting, Macrophages M1, and Macrophages M2).

As shown in Figure 6A, the relative scale of fraction of Plasma cells, T cells CD8, T cells CD4 memory activated, T cells follicular helper, T cells regulatory (Tregs), and NK cells activated was significantly up-regulated in high N-Scores group compared to low N-Scores group (*p* < 0.05). Additionally, T cells regulatory (Tregs) showed an obviously positive correlation with N-Scores (coef >0.25). By contrast, T cells CD4 memory resting, Macrophages M1 and Macrophages M2 had a remarkably negative correlation with N-Scores (coef < −0.25) (Figure 6B). The correlation scaler plot further demonstrated the relations between these immune cells and N-Scores (Figure 6C).

### N-scores contributed to immunotherapy and molecular drug treatment

**FIGURE 7:**
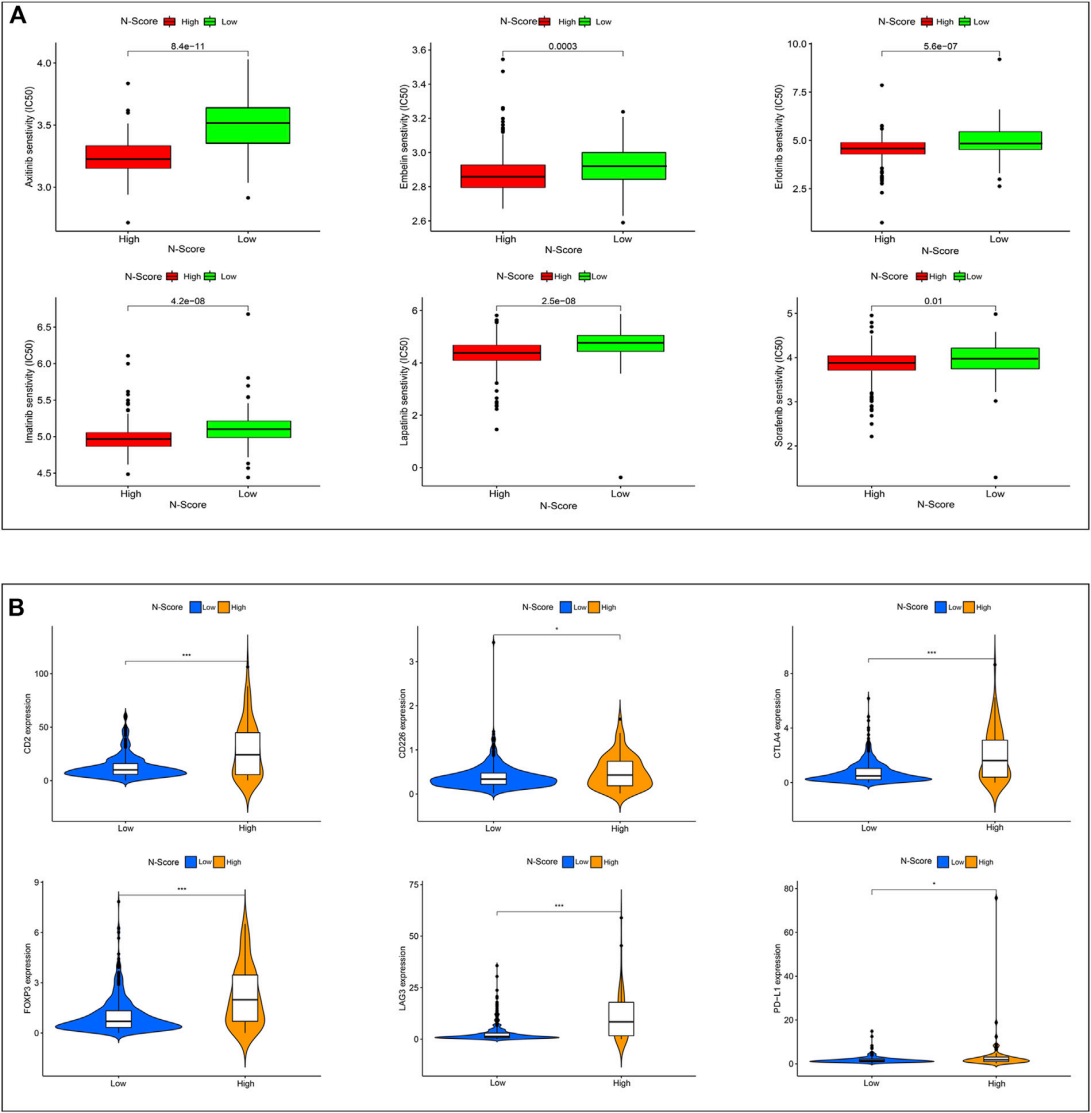
Effect of N-Score in the therapy of ccRCC patients. **(A)** The molecular response of two N-Score groups for six common molecular drugs (Axitinib, Embelin, Erlotinib, Imatinib, Lapatinib, and Sorafenib). **(B)** The expression of six immune checkpoint molecules (CD2, CD226, CTLA4, FOXP3, LAG3, and PD-L1) in two N-Score groups (*: *p* < 0.05, **: *p* < 0.01, and ***: *p* < 0.001).

Figure 7A showed the results of drug sensitivity test between N-Scores and six commonly used molecular drugs (Axitinib, Embelin, Erlotinib, Imatinib, Lapatinib, and Sorafenib) targeting ccRCC. The result demonstrated that the IC50 of all molecular drugs was remarkably lower in the high N-score group, indicating that the high N-Score group was more sensitive to all molecular drugs, which was consistent with the general law of drug sensitivity treatment. Further studies were performed to explore the relations between immune checkpoint expressions (CD2, CD226, CTLA4, FOXP3, LAG3, and PD-L1) and N-Scores (Figure 7B). In the iMvigor210 cohort, the association between N-score and anti-PD-L1 response was further explored (Supplementary Figure S2D). These results revealed that the immune checkpoint expressions of the high N-Scores groups were increased, indicating that they may be better treated with ICI treatment.

### External clinical application value of N-scores

**FIGURE 8:**
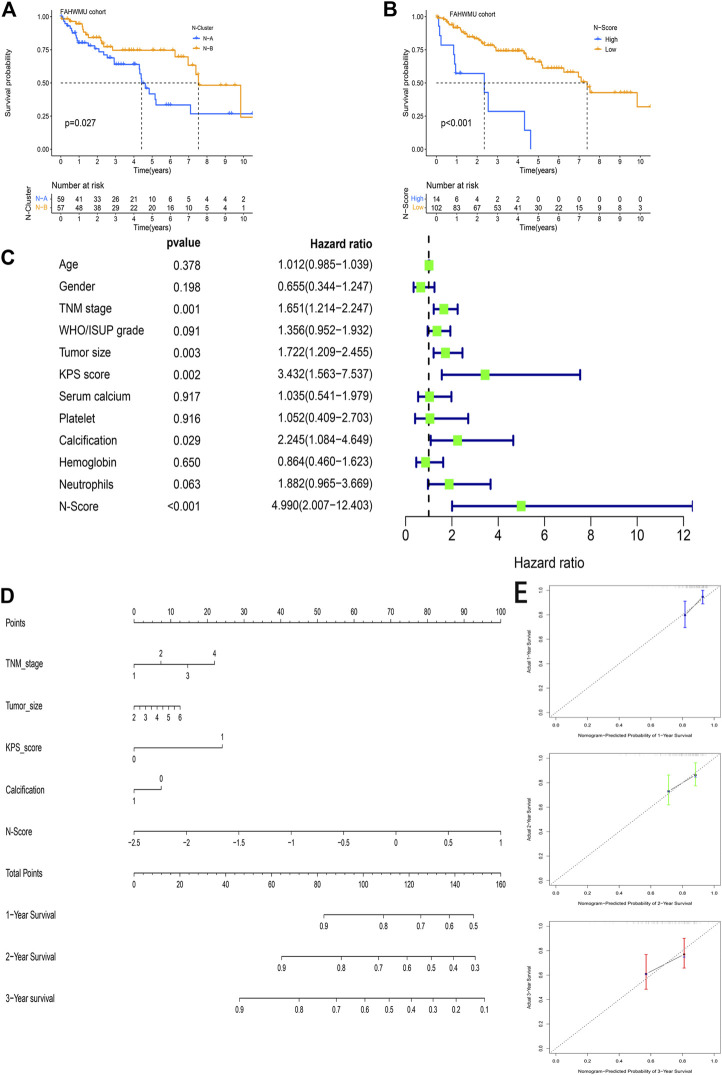
The external clinical value of the N-Score. **(A)** Kaplan-Meier (K-M) survival curve shows that the effect of N-Clusters on overall survival (OS) in the FAHWMU cohort (*p* < 0.027). **(B)** Kaplan-Meier (K-M) survival curve shows that the effect of N-Scores on overall survival (OS) in the FAHWMU cohort (*p* < 0.001). **(C)** The univariate Cox analysis of N-Score and clinical characteristics (Age, Gender. TNM stage, WHO/ISUP grade, Tumor size, KPS score, Serum calcium, Platelet, Calcification, Hemoglobin, and Neutrophils). **(D)** The nomogram combining TNM stage, Tumor size, Calcification, and N-Score. **(E)** The calibrate curve on the 1st, 2nd, and 3rd years.

In the FAHWMU cohort, N-Clusters were identified based on the consensus clustering method above (Supplementary Figures S1C,D). According to the PCA analysis, N-Scores were also obtained. The K-M survival curves indicated the OS difference between N-Clusters and N-Scores, which was similar compared to the results in the TCGA cohort (Figures 8A,B, *p* < 0.05). To further explore the deep prognostic value of N-Score, the univariate Cox analysis was performed among N-Score and clinical characteristics (Age, Gender, TNM stage, WHO/ISUP grade, Tumor size, KPS score, Serum calcium, Platelet, Calcification, Hemoglobin, and Neutrophils) (Figure 8C). It was found that N-Score could be served as an independent risk factor to predict the prognosis of ccRCC (Hazard ratio = 4.990, 95% CI = 2.007–12.403, *p* < 0.001). Meanwhile, several clinical characteristics (TNM stage, Tumor size, KPS score, and Calcification) also showed the similar clinical value (*p* < 0.05). Subsequently, a novel clinical nomogram was constructed to determine the combined value of N-Score and theses clinical characteristics (Figure 8D). It could be indicated that N-Score showed higher sensitivity and prediction efficiency than other indicators. The calibrate curves verified the robust effect of the nomogram in the 1st, 2nd, and 3rd years (Figure 8E). The DCA analysis further demonstrated that the role of N scores in clinical decision-making is efficient in the 1st, 2nd, and 3rd years (Supplementary Figures S2E–G).

## Discussion

As a routine method for clinical diagnosis, the AJCC staging system is widely used in the decision-making and monitoring of ccRCC (Alexandrescu et al., 2020). However, due to the limitation of ccRCC clinical heterogeneity, the AJCC systems have not been able to guide treatment with complete precision (Suss et al., 2021). At present, with advances in immunology and precision medicine, the modalities for treating ccRCC have become diverse (Cowman et al., 2020; Kim et al., 2021). For instance, it has been reported that ICI treatment (e.g., pembrolizumab, lenvatinib) and molecular drugs (e.g., benzo [4] helicenium, Telaglenastat) could serve as key targets for targeted therapy of ccRCC (Lee et al., 2021b; He et al., 2021; Meric-Bernstam et al., 2022). Meanwhile, multiple regulatory pathways of apoptosis have also been confirmed to remarkably influence the carcinogenesis and immunotherapy of ccRCC (Colli et al., 2021; Yang et al., 2021). All the above treatment options contribute to providing effective treatments for RCC. However, they also imply that more precise and stable evaluation methods are needed to assist in performing clinical decisions. Actually, robust evaluation biomarkers, that can precisely identify the prognosis of ccRCC patients, who may be benefited from ICI treatment, molecular drugs targeted and TMB assessment, are still lacking. To make up this shortcoming, we attempt to investigate the correlations between necroptosis and the prognosis, TMB characteristics, immune response and drug sensitivities of ccRCC.

It has been reported that the identification of ccRCC subtypes by gene expression profiling facilitates the accurate discrimination of patients’ prognosis (Verbiest et al., 2018; Linehan and Ricketts, 2019). It lays the foundation for the development of improved methods to diagnose, treat, and prevent this disease. Notably, N-clusters identified in our study can precisely identify the different survival status and differences in tumor microenvironment infiltration of ccRCC patients. Currently, genetic markers associated with necroptosis have been constructed in various cancers (e.g., Hepatocellular carcinoma and breast cancer) (Hu et al., 2022; Yang and Jiang, 2022). Additionally, Xie and others constructed a powerful necroptosis index to accurately predict prognosis in triple-negative breast cancer (Xie et al., 2022). But unfortunately, these genetic markers were constructed through public datasets and lacked suitable external cohorts to conduct in-depth exploration of the validity. In this work, N-Scores showed the superior value in the prognosis prediction and targeted treatment both in the TCGA cohort and FAHWMU cohort. Moreover, the superiority of our N-scores was further reflected in clinical application. As indicated in the results, the N-scores could combine TNM stage, Tumor size, KPS score, and Calcification to achieve the efficient prediction of prognosis in the FAHWMU cohort. These results largely compensate for the inadequacy of similar studies.

Currently, traditional clinical indicators (e.g., AJCC staging system, degree of invasion, tumor size, etc.) are still conveniently used in the outcomes and decision-making of ccRCC (Turajlic et al., 2018). The application of emerging biomarkers (e.g., TMB, TP53, ICB, and microsatellite state) are also significantly correlated with the targeted treatment of ccRCC (Au et al., 2021; Krishna et al., 2021). Meanwhile, the use of emerging molecular agents for adjuvant therapy has also become a key approach in RCC treatment (Choueiri et al., 2021). Notably, N-Scores in this work also showed superior performance in prognosis prediction of ccRCC. Validated by the FAHWMU cohort, it revealed the potential value as a precision biomarker to assess the prognosis of ccRCC compared to other clinical factors. Moreover, our results demonstrated that the increase of N-Scores can be negatively associated with the increase of the relative content of macrophages and T cells CD4 memory resting. It was consistent with the previous studies targeting T cells and macrophages infiltration characteristics in ccRCC (Krishna et al., 2021). Moreover, the N-Scores played a key role in the assessment of immunotherapy characteristics. In addition, as a highly frequently mutated gene in ccRCC, VHL has been confirmed as the key indicator for evaluating cancer metastasis and therapeutic efficacy (Yao et al., 2017; Hsieh et al., 2018). Notably, N-Scores in this work can precisely predict the mutation levels of VHL, which may help assessing the cancer progression of ccRCC. Currently, it has been found that cabozantinib in combination with atezolizumab demonstrated encouraging clinical activity and acceptable tolerability in patients with advanced ccRCC (Pal et al., 2021). Actually, numerous molecular drugs have also been invested in the treatment of ccRCC (McDermott et al., 2021). In this respect, our N-Scores showed the significant value in predicting the sensitivity of molecular drugs, which may contribute to the relevant drug treatment.

The advantages of this study could be summarized in the following aspects. It is the first time to develop the N-Scores to improve the prognostic outcomes of ccRCC. Moreover, N-Scores contribute to the evaluation of immune infiltration characteristics, tumor mutation levels, and treatments sensitivities for ccRCC patients. In addition, the clinical implication of N-Scores was further validated in the FAHWMU cohort. Our nomogram further combined TNM stage, Tumor size, KPS score and Calcification to achieve the efficient prediction of prognosis.

In conclusion, based on the combination of bioinformatics and external clinical validation, we develop the robust and powerful N-Scores for assessing the prognosis, tumor immune infiltration characteristics, tumor mutation levels and treatments sensitivities of ccRCC. This N-Score may contribute to improving outcomes and decision-making for ccRCC patients.

## Data Availability

The original contributions presented in the study are included in the article/Supplementary Material, further inquiries can be directed to the corresponding authors.
