# Supplementary figures and images for: Development and validation of a novel necroptosis-related score to improve the outcomes of clear cell renal cell carcinoma
Source: Front Genet. 2022 Sep 12;13:967613. doi: 10.3389/fgene.2022.967613 (PMC9510770; doi:10.3389/fgene.2022.967613)

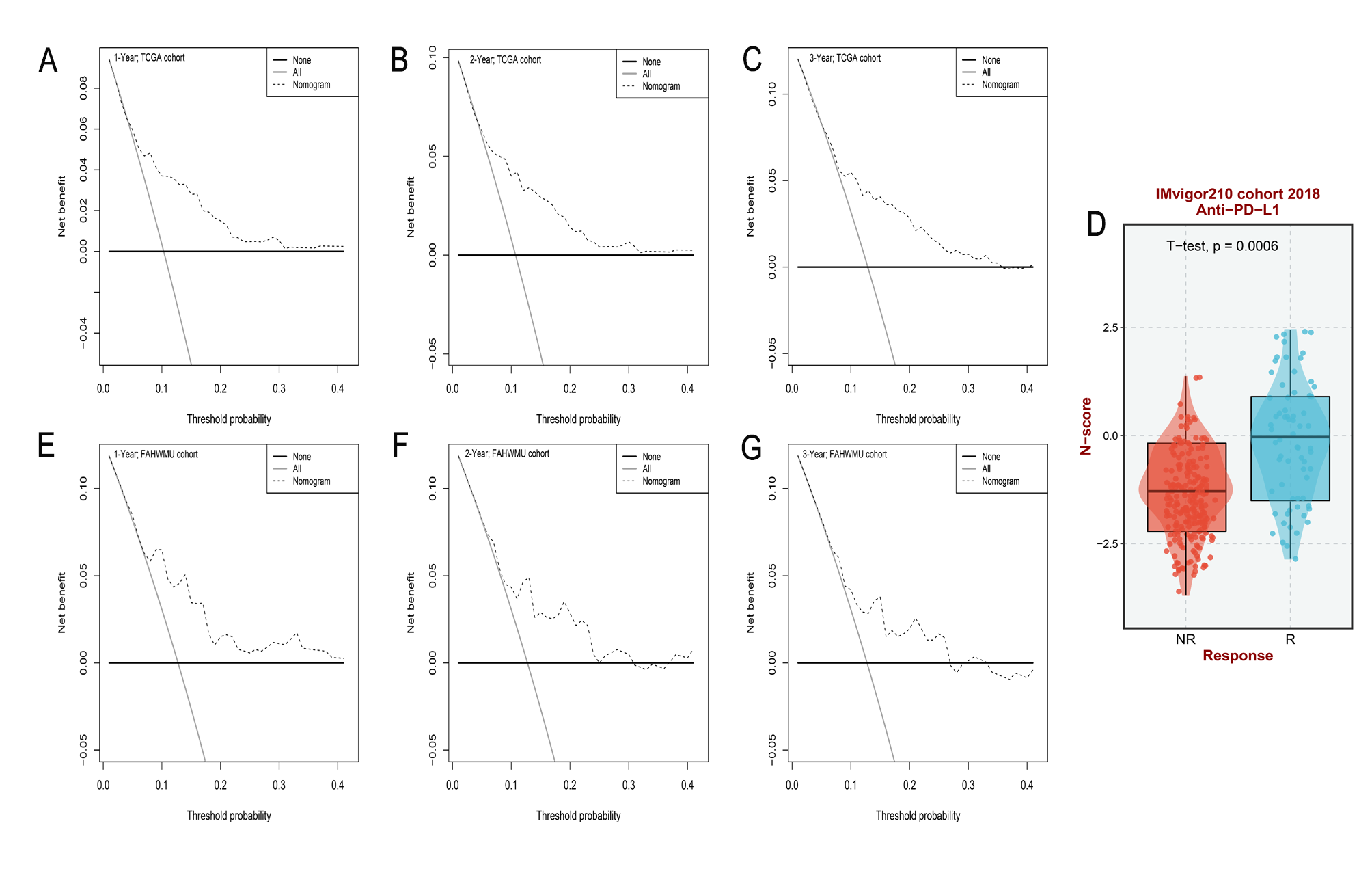

Supplement: Supplementary file 1 [file Image2.TIF]

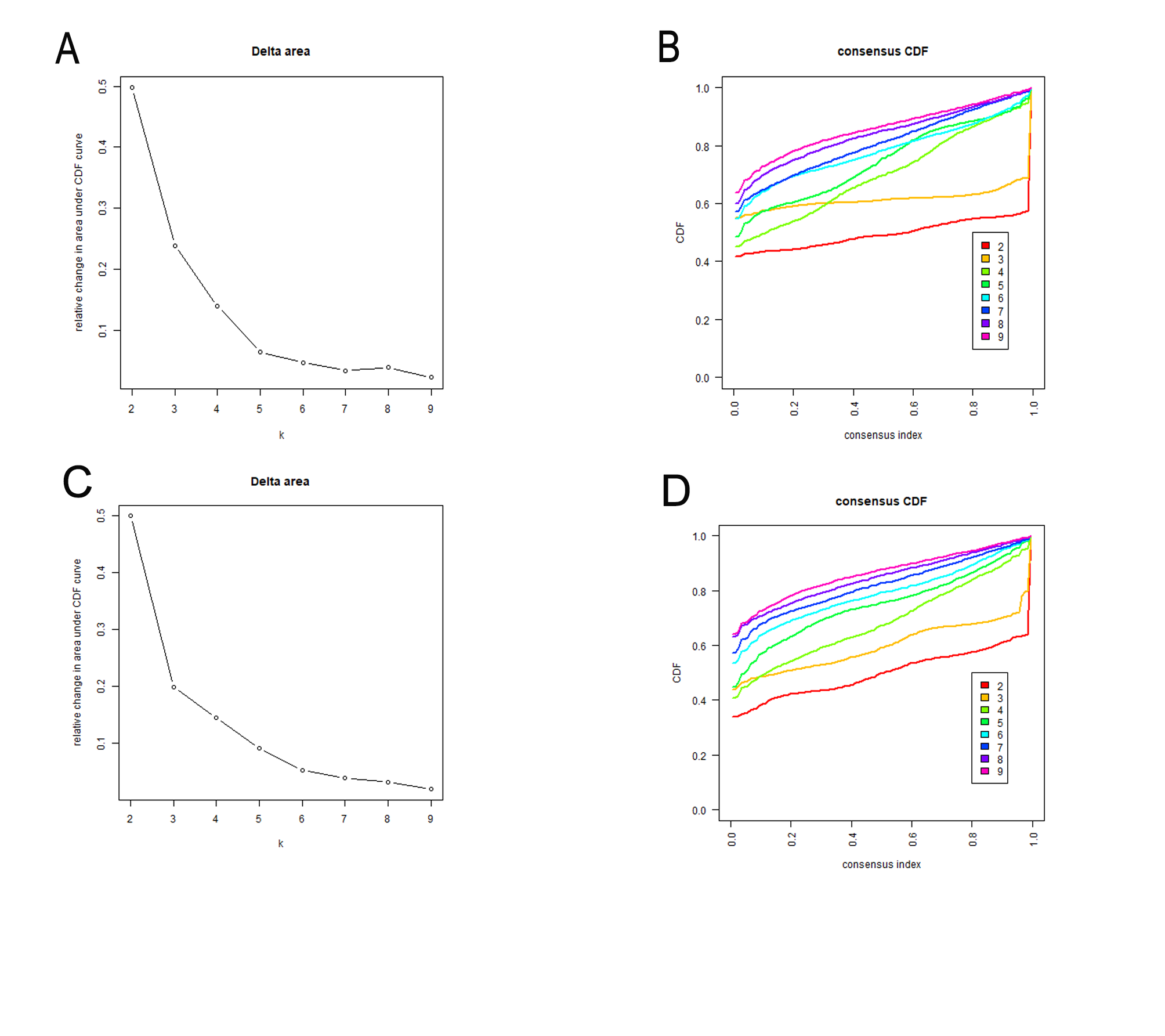

Supplement: Supplementary file 2 [file Image1.TIF]
